# Historical and current distribution ranges of the Asiatic black bear (*Ursus thibetanus*)

**DOI:** 10.1038/s41598-024-51588-5

**Published:** 2024-01-30

**Authors:** Unza Waqar, Tariq Mahmood, Muhammad Mushtaq, Ghulam Murtaza, Muzna Kashaf, Ayesha Sheraz

**Affiliations:** 1 Department of Zoology, Wildlife and Fisheries, PMAS-Arid Agriculture University Rawalpindi, Rawalpindi, 46300 Pakistan; 2 Expert Remote Sensing and GIS, House # 01, Street # 21, F8/2, Islamabad, Pakistan; 3 Hagler Bailly Pakistan, Islamabad, Pakistan

**Keywords:** Ecology, Zoology

## Abstract

The current distribution of Asiatic black bear (*Ursus thibetanus*) is available on the IUCN Red List of Threatened species website; however, nothing is known about the historical extent and occurrence of this species. Therefore, we aimed to understand the historical distribution of the Asiatic black bear, and map and estimate its total size, to compare it with that of species current distribution. In addition, we analyzed a network of protected areas in the past and current ranges of the species. We employed geographic information system (GIS) software to reconstruct and measure the historical range of the Asiatic black bear, comparing past and current ranges to analyze its expected range contraction. The main focus of the study was to enhance our understanding of the species' historical distribution, contributing to better conservation strategies for the present and future perspectives. The utilization of GIS tools facilitates a comprehensive exploration of the factors influencing the species' decline, ultimately aiding in more effective management and conservation efforts. We used published records of black bear’s occurrence in anywhere in history to reconstruct its historical distribution range. Results revealed that the Asiatic black bear was more widely distributed in historical times and its range spanned across approximately 15.86 million km^2^ while its current range is limited to approximately 7.85 million km^2^, showing a range contraction of approximately 49.5% (8.02 million km^2^ reduced). The total protected areas in the historical range of the species were found to be N = 9933, with total size of 0.946 million km^2^, against N = 6580 (0.667 million km^2^) that are present in the current range. Approximately 27.5% of the protected areas have lost the Asiatic black bear since historical times.

## Introduction

Large carnivores are considered as conservation tools to measure and avoid the loss of biodiversity because they are frequently used as flagship, umbrella, or indicator species^1^. These large carnivores also help in maintaining the equilibrium of ecosystems^2^. However, the challenge in their conservation persists, partly due to a restricted understanding of the intricate spatial dynamics essential for ensuring population viability^3,4^. The habitat requirements of such species deserve particularly close attention because they generally require large home ranges, are negatively impacted by changes in land use, and are killed because of the threats they pose to livelihoods These large carnivores also help in maintaining the equilibrium of ecosystems^2^.

The Asiatic black bear (*Ursus thibetanus*), being a top predator, has diverse foraging habits and consumes a variety of foods, such as nuts, insects, ungulates, and carrion^2^. Since bears are omnivorous and voracious^5^, they frequently eat fruit orchards, farmed fields of maize (*Zea mays*), and other agricultural places. Being scavengers, bears play a vital role in preventing the spread of illnesses and pests by consuming rotting food, especially animal corpses^6^. Black bears exhibit a notable capacity to adapt to diverse environments and their ability to thrive in a range of settings. Their dens are often easily accessible, which can pose challenges for wildlife conservation efforts^7^, because poachers can easily target them. This accessibility can result in disturbances, leading to stress, displacement, or harm to the animals and posing obstacles for wildlife conservation efforts^7^.

Compared to other bear species, the Asiatic black bear exhibits more aggression toward people. Numerous individuals, including hunters, wildlife managers, and local herders, are intrigued by Asiatic black bears (as poachers), which frequently encounter human–wildlife conflict due to their versatility in diet and adaptability to various habitats^8^. When food is low in the late autumn and early winter, Asiatic black bears display increased assertiveness and venture into locations they wouldn't typically frequent. This behavior leads to attacks on livestock as they seek animal protein^9^. Although their typical diet consists of grubs and insects, bears have few natural predators in their environment, but one of their main threats comes from Siberian tigers, which prey upon them who may kill them if they are seen dining on carrion. Asiatic black bears have been observed attacking sheltered household animals at night and hunting farmed animals in grazing areas^9,10^ .

The medium-sized Asiatic black bear is found in Central to South East Asia, as well as Japan^11^ . Over the past 30 years, populations of this species have decreased by 30–40%. The habitat of Asiatic black bear appears to be fragmented, and there is a noticeable trend of population decline^12^. The species is known to inhabit an extensive array of habitats, spanning from boreal forests to tropical rainforests in East to Southeast Asia^12^. Due to the human conflict with rural residents that live close to its favored habitats, the Asiatic black bear receives greater public attention when contrasted with certain other large mammals in Asia^13^. Although it is illegal to hunt black bears in most of South–east Asia, there is a huge incentive for poaching due to weak law enforcement and the high value of the species gall bladder, paws, and cubs. Additionally, Asiatic black bears are ruthlessly slaughtered to cater to the black market's demand for their paws, considered an exotic delicacy, and their gall bladders (bile), which are utilized in Traditional Chinese Medicine^14^. Poachers primarily target the black bear due to its diverse diet and adaptability to different habitats, contributing to animal-human conflicts^8^. This focus on U. thibetanus raises questions about whether it is the preferred target compared to other bear species, given that many Ursus species share similar diversity in diet and habitat. Human–Wildlife conflict, a global issue affecting various geographic and demographic circumstances, is intricately linked to such targeted activities^15^.According to a recent IUCN range map published (2020), the Asiatic black bear habitat is only found in a few provincial and national protected areas^16^ due to its local extinctions in some of its geographical areas^17^ and habitat destruction^18^. However, this range map only describes the area occupied by the Asiatic black bear and it does not distinguish between occupied and unoccupied habitats. Because many species' habitats are altered due to climate change, their distribution patterns are also shifting, which increases the likelihood of conflict between humans and wild animals^19^. The expansion of appropriate habitats outside the limits of protected areas may shift because of climate change^20^. Due to their extensive distribution, low population size, and increased human pressure, habitat loss and fragmentation have a particularly significant impact on large carnivores such as bears, making them especially vulnerable^21–23^.

An increased understanding of Asiatic black bear distribution and relative abundance, as well as a repeatable method of population monitoring throughout time, are necessary for its effective conservation^24^ which requires understanding a species' preferred habitat. To ensure the proper management and protection of a species, it is crucial for managers to be acquainted with the type and condition of the habitat where it thrives^25^.

The sustainability of environmental variables is threatened due to increased human manipulation of natural resources and the environment, particularly climate change^26^. Today, protected areas are seen as the last remaining heaven for animals, especially the large megafauna. Currently, a major global concern is climate change, which detrimentally impacts the boundaries of protected areas and the optimal distribution of habitats for various species^27,28^ with the expectation that it will also influence numerous organisms across different yecosystems, including several mammals facing the threat of endangerment. Ali et al.^29^ therefore, it is expected to influence the distribution ranges of large mammals, especially. Asiatic black bears are prone to exhibiting larger home ranges as large animals have larger home ranges^10^. The conservation of this species raises significant concerns, encompassing habitat limitations, degradation, and anthropogenic causes. Given their frequent interactions with humans, black bears are particularly vulnerable to the adverse effects of habitat fragmentation, degradation, large home ranges, and small population sizes^30^.

At current, the Asiatic black bear distribution spans across central, eastern, and southeast Asia. But in numerous countries within its range, the species faces conflicts with humans, leading to a decline in its population. The black bear, found in diverse habitats ranging from evergreen oak forests to grasslands^31,32^, faces a significant threat in Asia especially poaching for illegal markets^18,33^.

To date, no scientific studies have documented the past or historical occurrence and distribution range of the Asiatic black bear. The historical distribution range of the species remains undocumented, and there are no estimates available for comparison with its current distribution. This absence of historical records hinders our ability to assess whether the species' range has contracted or expanded over time. Therefore, the main objective of the current study was to reconstruct and map the historical or past distribution range of the Asiatic black bear. Additionally, the study aimed to measure the size of the reconstructed historical distribution range of the species and then compare it with its current distribution range. In addition, we aimed at investigating the network of Protected Areas, present in the past and current distribution ranges of Asiatic black bear.

## Methods

### Study Area

The Asiatic black bear (*Ursus thibetanus*) is indigenous to Asia occurring in different countries of the region except Malaysia. The study encompassed the entirety of the Asian continent, with a specific focus on documenting the historical existence of Asiatic black bears in any location within Asia.

### Study design

The current study aimed at investigating the historical distribution range of the Asiatic black bear and comparing it to its current distribution range. Data about the past occurrence of Asiatic black bear were collected from both published and unpublished sources such as peer-reviewed research articles of scientific journals, books, newspapers, magazines, as well as unpublished theses etc. We conducted our literature search using internet search engines such as Google and Google Scholar. Additionally, we utilized web resources like the Global Biodiversity Information Facility (GBIF) website (https://www.gbif.org/) for published literature about the historical occurrence of the Asiatic black bear. The full text articles were downloaded and saved, and historical locations of the Asiatic black bear mentioned were identified, marked, and fed into “Google Earth Pro for desktop” software, specifically version 7.3 (you can find it at https://www.google.com/earth/about/versions/). Then we compiled these locations into an Excel spreadsheet (Supplement material) and pinpoint them on a map so that their geographic coordinates (Latitudes and Longitude) were retrieved and saved as keyhole Markup Language (KML) file, which were later converted and saved as shape file for further analysis. These location data were then imported to QGIS desktop software for processing and analysis. During analysis, the historical distribution of the black bear was reconstructed and quantified and mapped. Likewise, the current spatial data about the distribution of black bear was retrieved from the IUCN Red List of Threatened Species^12^ and analyzed for measuring the current range size. The spatial data about the continent's Protected Areas was downloaded from the website of "Protected Planet" and analyzed to study about which protected areas have lost the black bear species since historical times. In the end, the historical and present distribution ranges of Asiatic black bear were compared to investigate any range contraction or expansion.

### Data collection

The data about the historical occurrence of the black bear on the globe, were collected by searching on internet for a variety of published resources, including research articles in the peer reviewed scientific journals, books, scientific magazines, newspapers, and unpublished dissertations as well. We defined "historical distribution/occurrence” of Asiatic black bear as a species’ regular occurrence in the past at any time during history in the previous centuries, and all the data till current distribution of the species was included in the analysis. Mainly due to lack of published old records, we could find oldest published records of the species till 1826. Data on ecological parameters of the species were also obtained such as habitat or vegetation type, altitude of species occurrence, home range size and population. The presence of the target species outside of its current region of distribution were recorded along with information on the duration of its presence, the reliability of the evidence (either it was based on paleontological discoveries, hunting, or information from the neighborhood first-hand accounts) and other pertinent ecological data (altitude, vegetation type when available). However, the information that was not supported by any reliable evidence was discarded and excluded from the current analysis.

Likewise, the spatial data about the current range of the Asiatic black bear were retrieved from the International Union for Conservation of Nature's website (IUCN Red List of Threatened Species) for use in the ongoing analysis^12^, This was basically performed to compare the current range of the species with that of its historical range.

We also retrieved spatial data on the protected areas of the world from the “World Data on Protected Areas” (WDPA) using the website “Protected planet” (http://www.protectedplanet.net) for their analysis in the historical and current ranges of the Asiatic black bear.

### Data processing and reconstruction of historical range

Data collected about past occurrence of the black bear species on the globe were entered into an Excel spread sheet, with all supporting evidence and information such as location, habitat type, diet, home range size, elevation and hunting and other threats. The species' location data were input into the "Google Earth" desktop software, to generate a KML (Keyhole Markup Language) file. This file was subsequently imported to QGIS (Quantum GIS Development Team, 2012) desktop software and transformed into a ‘shape’ file format to acquire the latitude–longitude geographical coordinates of the occurrence places of the black bear. The outcome was a layer of points where the species was historically distributed or known to exist. proceeded to construct polygon buffers surrounding our designated location points using QGIS. These buffers were configured to have a standardized size of 100 km^2^. To achieve this, I set the buffer distance parameter to 3.3°, a value chosen to closely approximate the desired 100 km^2^ area. The next step involved utilizing the "Buffer Dissolve" tool within QGIS. Here, the buffers were dissolved, and it's worth noting that the dissolution process maintained the same size parameter of 3.3°, as initially specified. And then the spaces or gaps between the buffers were filled using the option of ‘toggle editing’ and ‘add feature’ in the QGIS to get a polygon of the historical distribution range of the species^34^

Then utilizing "global topographical data," obtained from the website http://www.webgis.com/terr_world.html, we mapped earth’s surface (as globe) using GIS software , and the non-terrestrial areas (that is, major inland aquatic systems) of the buffers were clipped away and excluded from the analysis. Finally, we used the “toggling editor” option in the QGIS to fill in any gaps in the resulting ranges using historical locations and ecological factors. Quantification of the complete historical range of the black bear was carried out using the option of “Analysis tools, and Basic Statistics” in the same QGIS software after the historical distribution range polygon was reconstructed and then finally mapped. This reconstructed polygon represented the historical range of the Asiatic black bear.

During the geographical analysis, various spatial operations were applied to the map, notably a buffer dissolved that merged overlapping buffers to create a unified area. Subsequently, the resulting buffers underwent a clipping process, precisely defining the study area within a specific boundary or extent of interest. To ensure a focus solely on terrestrial land masses, non-terrestrial elements, such as water bodies were thoughtfully excluded or excised from the dataset. This careful exclusion aimed to isolate and concentrate exclusively on the terrestrial landscape, effectively removing the influence of non-terrestrial features.

### Data on current distribution range

For retrieving spatial data as polygon on the current distribution of the Asiatic black bear, the IUCN's Red List of Threatened Species^12^ website was used. Given the recent publication of this data (IUCN, 2020), the "current range" of our species, the black bear, represents the most precise information available concerning its present distribution. To define the extent of the black bear's present distribution area, the spatial data sourced from the IUCN Red List of Species website were analyzed for size and protected area estimation using QGIS desktop software,

### Comparison between historical and current ranges

The reconstructed historical distribution range of Asiatic black bear was measured for its size and estimated from the reconstructed distribution polygon and then compared with its current range published by IUCN^12^, to study the difference in sizes of the two distribution ranges and analyze the expansion or contraction of the distribution area of the black bear since historical times.

### Distribution of Asiatic black bear in protected areas

Within our designated study area, encompassing the Asiatic black bear's habitat range, a network of protected areas (PAs) was superimposed onto both the historical and current distribution ranges of the species. This was conducted to explore the number and size of PAs that existed in the species ‘historical distribution range as well as those currently present. The purpose was to identify and quantify the PAs that have experienced a loss of the black bear population since historical times. This process facilitated an examination of the count and proportions of PAs that existed in the species' historical and present distribution ranges Finally mapping was done.

## Results

### Historical distribution

Data collected about historical distribution of the Asiatic black bear revealed that the species was present in different countries: India, Pakistan, Nepal, Bhutan, Russia, China, Japan, Laos PDR, Korea, Thailand, Iran, Taiwan, and Vietnam, all of which had significant populations of the studied species. The oldest published record of occurrence of the Asiatic black bear found in India dates to the year 1826. Whereas the most recent distribution records can be found in the IUCN Red List of Threatened Species.

While gathering data, certain atypical distribution records were identified, including the one instance in Russia. But due to limited availability and lack of any concrete evidence and verification from other sources, these records were not included in the final analysis of the study. The study primarily focused on the Asian region due to the widespread distribution of the Asiatic black bear in that area.

**Figure 1:**
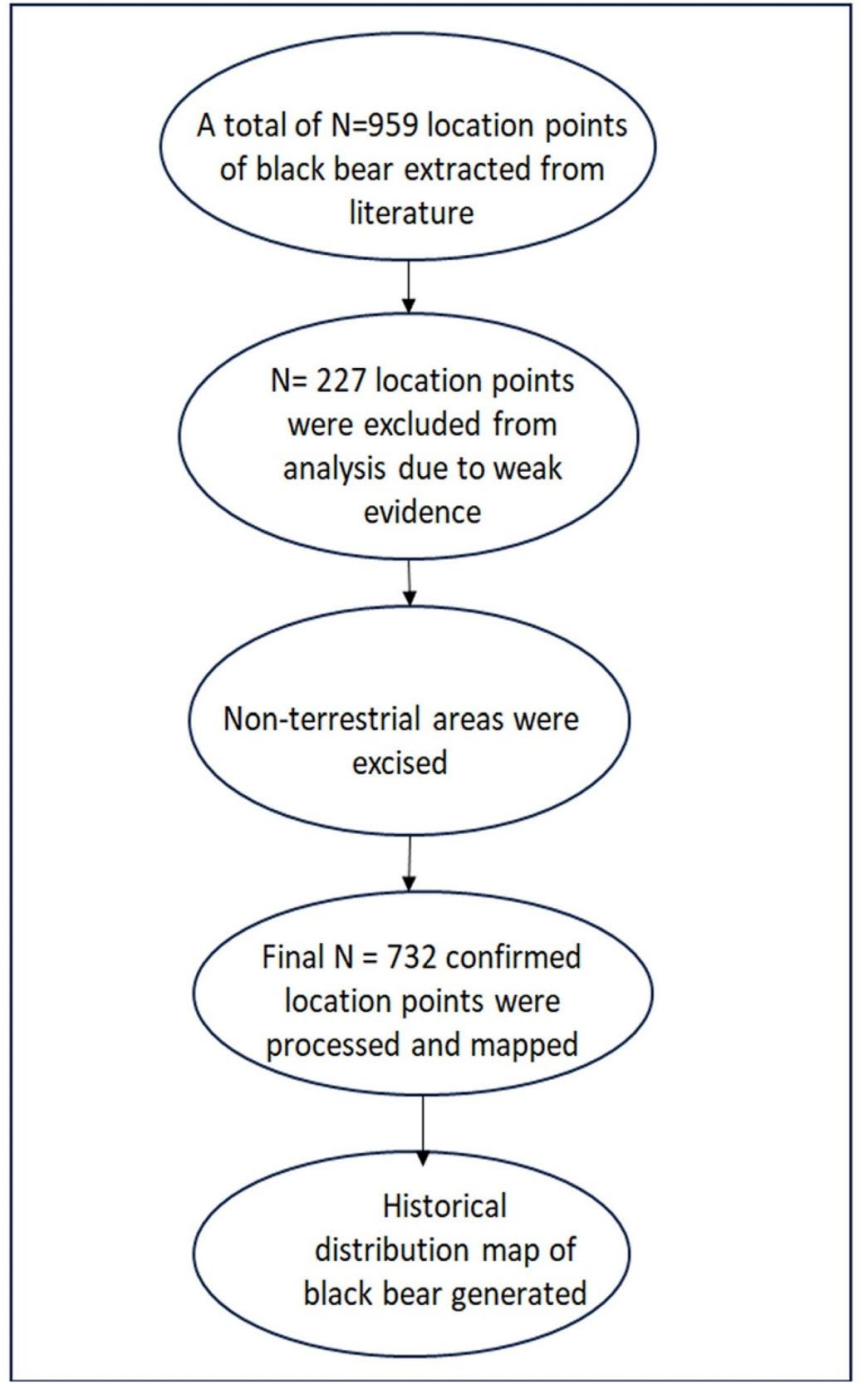
Details of historical records about Asiatic black bear that were found and analyzed.

Overall, a total of N = 959 location points were extracted, out of which N = 732 were processed for analysis. Duplicate information (N = 227 locations) and weak or non-terrestrial evidence were excluded from the analysis, resulting in the use of N = 732 locations for the final analysis (Fig. 1).

**Figure 2:**
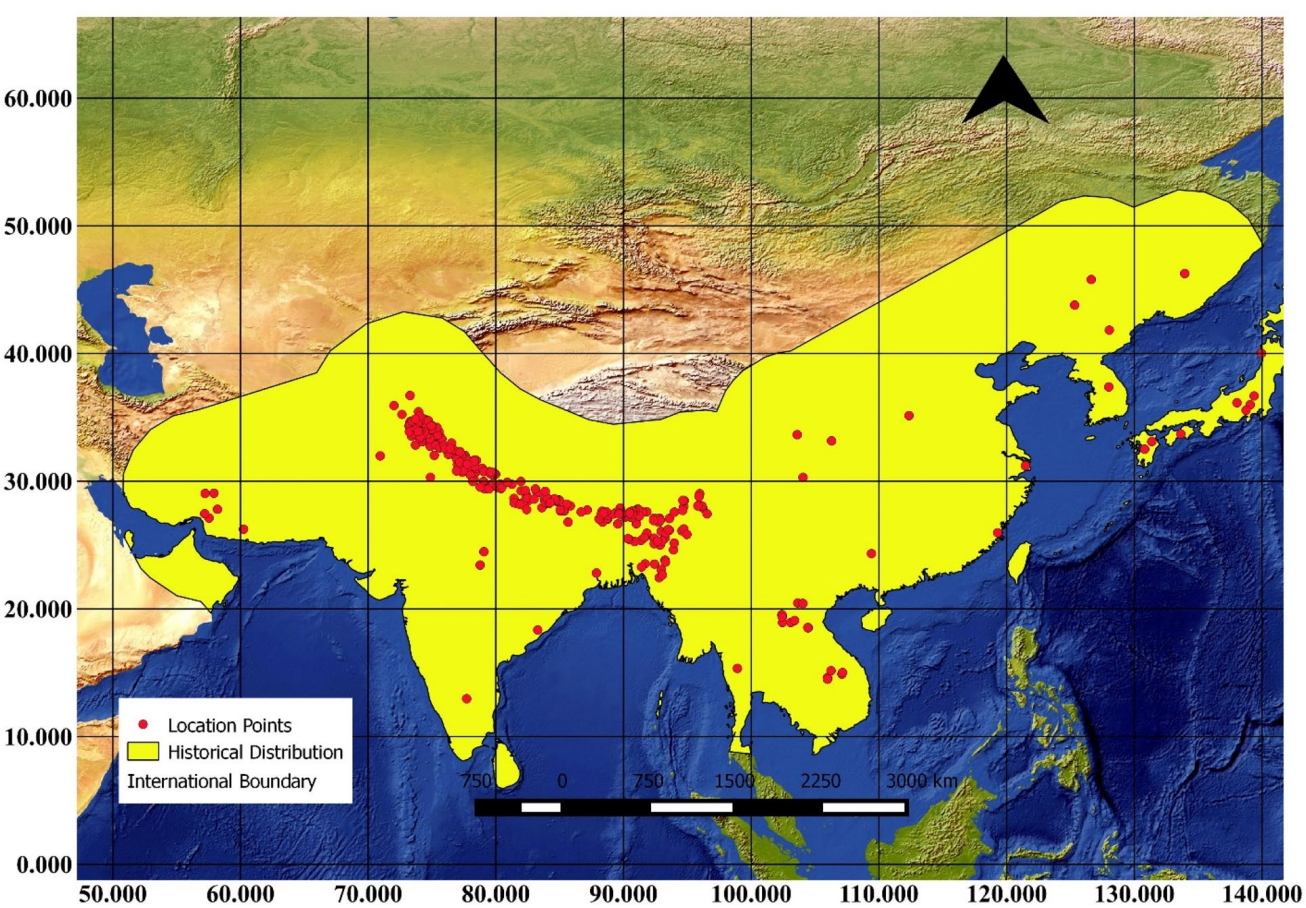
A GIS-based map showing the reconstructed historical distribution range of the Asiatic black bear along with location points, the distribution polygon was created to reconstruct the past range of the species and quantified in QGIS software.

The analysis of the historical distribution range revealed a broader geographical presence of the Asiatic black bear during historical times. The reconstructed past distribution range polygon of the Asiatic black bear was measured and found to be having a total area of approximately 15.86 million km^2^ (Fig. 2).

### Current distribution range

**Figure 3:**
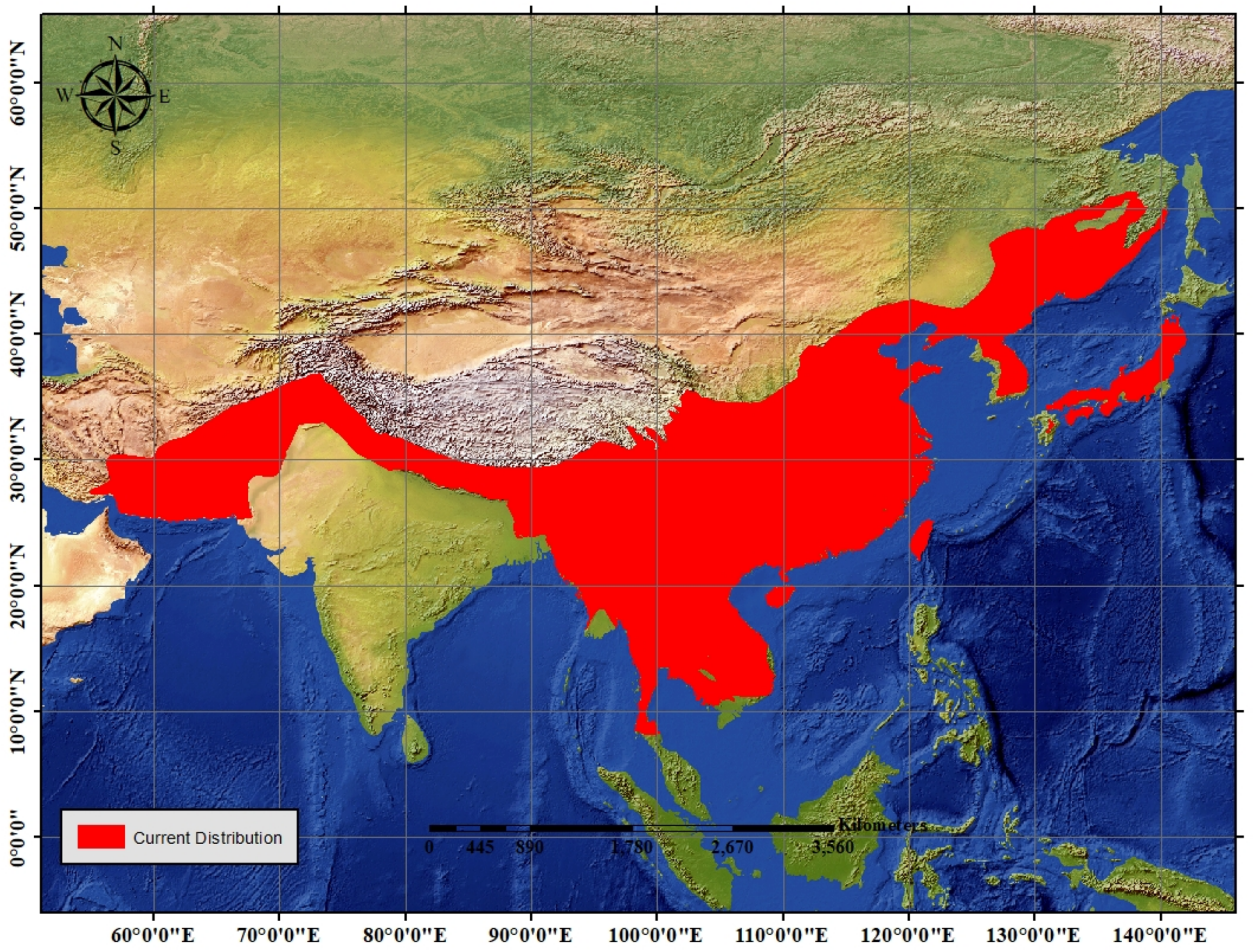
A map showing the current distribution range of the Asiatic black bear downloaded from IUCN Red List of threatened species (2016) and modified and utilized for quantification of the species range.

The current distribution range of the Asiatic black bear quantified revealed different countries having Asiatic black bear population at present. (Fig. 3), The total size of the current range of the species was quantified to be approximate an area of 7.85 million km^2^.

### Range contraction

**Figure 4:**
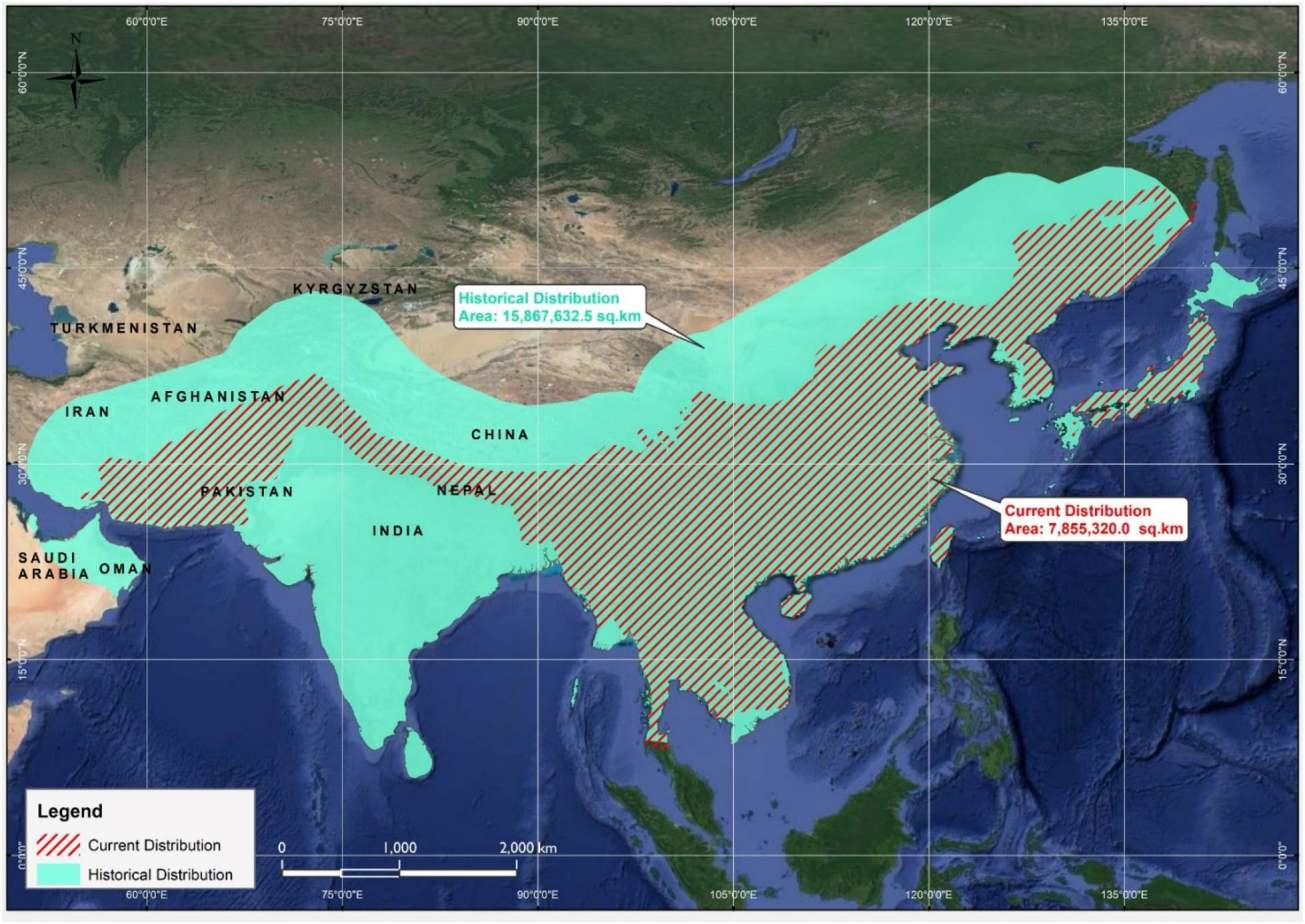
A GIS-based map showing comparison of the historical and current ranges of the Asiatic black bear along with the respective size areas of these two ranges.

The historical distribution range covered a more extensive area, spanning across 15.86 million km^2^ globally, whereas the current range is much shorter, measuring approximately 7.85 million km^2^. These data represent a substantial reduction or contraction of the Asiatic black bear distribution range, measuring approximately 8.01 million km^2^ area (49.5%), which the species no longer inhabits (Fig. 4).

### Protected areas and black bear range

The overall count of PAs in Asia, encompassing both terrestrial and marine ones, totaled N = 35,194. Among these, the Touran was distinguished as the largest protected area, covering an expansive size of 14,706.40 km^2^.

**Figure 5:**
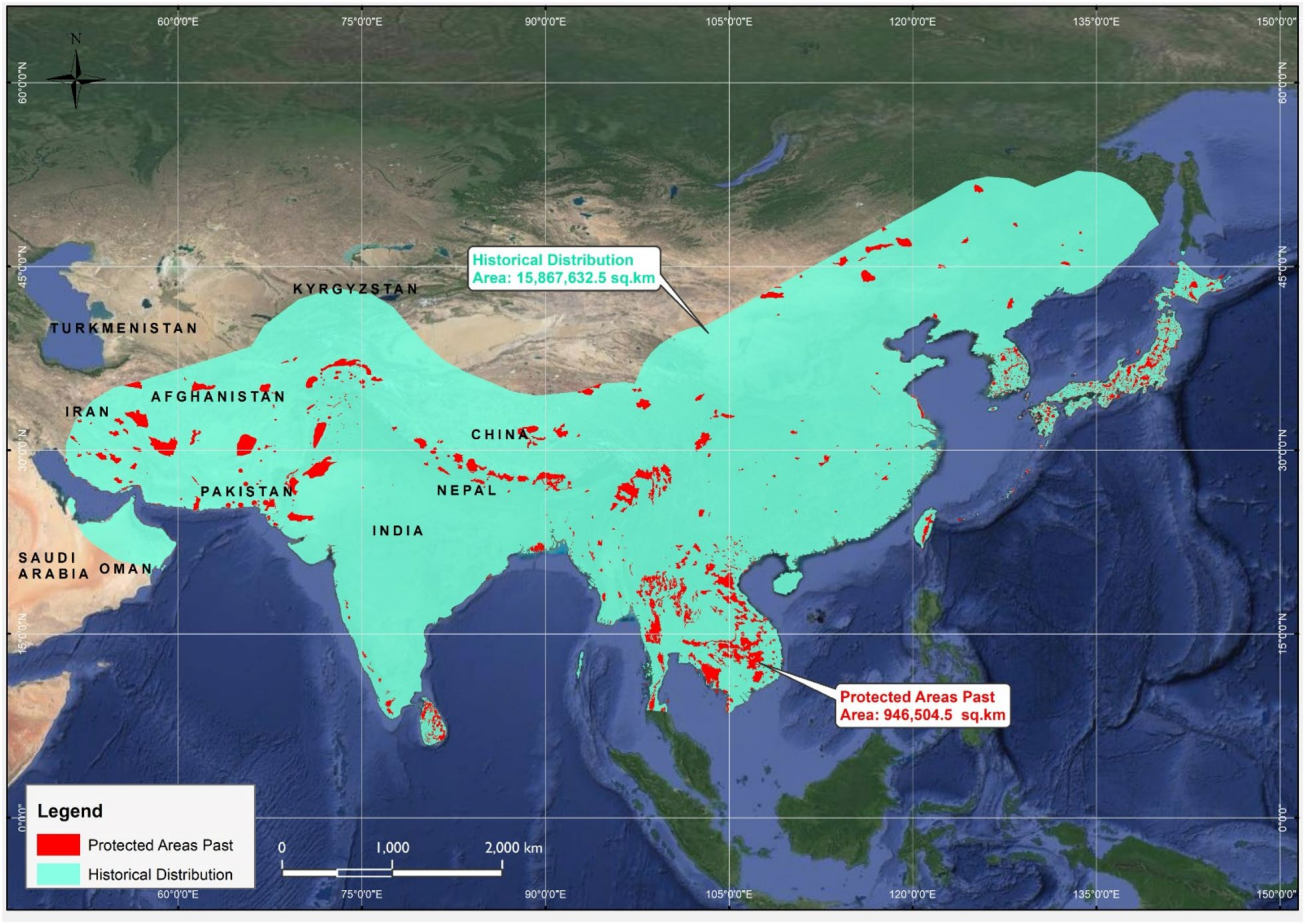
A Map showing the protected Areas (PA’s) of Asia that had Asiatic black bear in the historical range in the past.

The Spatial data (shape file layer) of protected areas overlapped or superimposed over the reconstructed historical distribution range of the Asiatic black bear revealed. a total of N = 35, 194 protected areas that were present spanning across the Asia, out of which a total of N = 9933 PA's were present within the historical distribution range of the Asiatic black bear (Table 1). Noteworthy, protected areas among those included Hugaung Valley Wildlife Sanctuary, Jigme Dorji National Park, Touran, Central Kravanh,Gando, and Western Nghe An and others (Fig. 5). Looking at the total size of the protected areas that were present in the historical range of the species, the data derived from ArcGIS revealed a total size of the protected areas being 0.95 million km^2^ (Fig. 5).

**Table 1:** Some of the major protected areas occurring in the past and current distribution ranges of the Asiatic black bear.

Sr#	Name of PA	Country	Category type	IUCN category	Status year	Area (km^2^)	Past range	Current range
1	Great Himalayan National Park Conservation Area	India	World Heritage Site (natural or mixed)	Not Applicable	2014	905.40	Yes	Yes
2	Hkakaborazi National Park	Myanmar	National Park and ASEAN Heritage Park	II	1998	3,812.4	Yes	Yes
3	Hugaung Valley Wildlife Sanctuary	Myanmar	Wildlife Sanctuary	IV	2004	6,371.3	Yes	Yes
4	Central Kravanh	Cambodia	National Park	II	2016	4,013.1	Yes	Yes
5	Kaev Seima	Cambodia	Wildlife Sanctuary	IV	2016	2,937.6	Yes	Yes
6	Daisetsuzan	Japan	National Park	II	1934	2,267.6	Yes	No
7	Khunjerab	Pakistan	National Park	II	1975	2,269.1	Yes	No
8	Wilpattu	Sri Lanka	National Park	II	1938	1,316.6	Yes	No
9	Thung Salaeng Luang	Thailand	National Park	II	1963	1,275.6	Yes	Yes
10	Touran	Iran	UNESCO-MAB Biosphere Reserve	Not Applicable	1976	14,70	Yes	No
11	Khuen Si Nakarin	Thailand	National Park	II	1981	2,053.4	Yes	Yes
12	Dadohaehaesang	Korea	National Park	II	1981	2,266.2	Yes	No
13	Jigme Dorji National Park	Bhutan	National Park	II	1974	4,374.0	Yes	Yes
14	Taninthayi Nature Reserve	Myanmar	Nature Reserve	Ia	1980	1,699.99	Yes	Yes
15	Dureji	Pakistan	Wildlife Sanctuary	IV	1972	1,782.5	Yes	Yes
16	Yushan	Taiwan	National Park	II	1985	1,031.2	Yes	Yes
17	Wangchuck Centennial National Park	Bhutan	National Park	II	2008	4,914.65	Yes	Yes
18	Xianghai	China	Ramsar Site, Wetland of International Importance	Not Reported	1992	1,054.6	Yes	No
19	Gando	Iran	Protected Area	Not Reported	0	4,413.0	Yes	Yes
20	Western Nghe An	Viet Nam	UNESCO-MAB Biosphere Reserve	Not Applicable	2007	13,03	Yes	Yes

**Figure 6:**
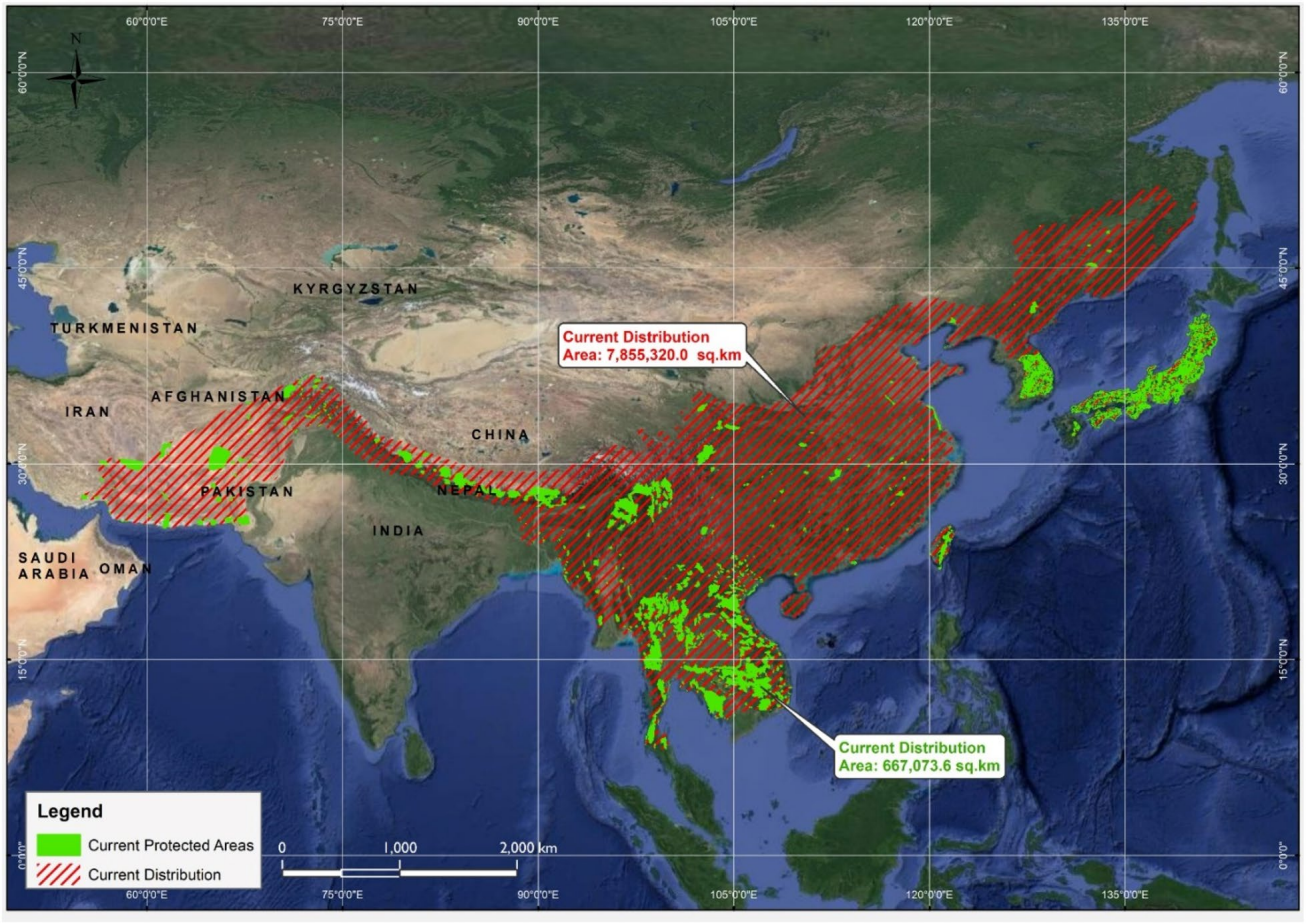
A GIS-based map showing protected areas (PA’s) of Asia (N = 6580) that are included in the current distribution range of the Asiatic black bear.

Similarly, for the current distribution range of the Asiatic black bear, the spatial layer of the network of protected areas was superimposed over the current distribution range of the species and clipped away. The results revealed that out of total of N = 35,194 protected areas (PA's) in Asia, only N2 = 6580 PA's are present in the current range of the Asiatic black bear (Fig. 6). Accordingly, the total size of the protected areas present in the current range of Asiatic black bear was estimated to be 0.667 million km^2^ (Fig. 6).

The analysis of the protected areas provides a comprehensive overview of approximately 35,194 such sites worldwide. This includes major protected areas that cover extensive land areas, as well as various common types that have been established throughout history and continue to be protected today. However, the analysis also reveals the unfortunate presence of information regarding protected areas that have lost the black bear species over time (Table 1, Supplementary material).

## Discussion

Most of the world's terrestrial large carnivores have undergone significant range contractions, and currently, many of these species are facing the threat of extinction. However, there has been limited effort to thoroughly quantify the extent of their range contractions, which hampers our understanding of the contributing factors behind these trends^35^. In this study, we have reconstructed the historical range of the Asiatic black bear and quantified it to its extent. By examining historical and current data, we reveal the size of range contractions that have occurred over time, and we hereby identify and report specific regions and protected areas where these contractions have been particularly pronounced. Over the past millennia, numerous species have faced significant declines attributed to the rapid influence of human activity and climate change. However, obtaining compelling evidence spanning such long-term time scales remains a challenge^35^ The current study has addressed this issue by searching and compiling historical records of the Asiatic black bear (*Ursus thibetanus*). Through an extensive literature review, data were gathered on species occurrences dating back to 1826.

To understand the factors influencing the range contraction of the Asiatic black bear, QGIS software was employed to quantify its distribution range over time, comparing historical and current data. In the specific context of India^36^, an approximate potential habitat range of 270,000 square kilometers for Asiatic black bears was reported, utilizing rule-based modeling in a Geographic Information System (GIS)."Specific information about the distribution range of the Asiatic black bear in Asia remained undocumented previously until our current study. This indicates that the Asiatic black bear has experienced a substantial range contraction of approximately 8.01 million sq km over time, reflecting habitat loss and reduction in distribution size. This significant decline highlights the challenges and threats faced by the species, underscoring the utmost importance of conservation efforts aimed at addressing and mitigating these range contractions to safeguard the habitat and population of the Asiatic black bear.

The percentage of reduction in the Asiatic black bear's distribution range is approximately 49.5%. The existing condition of wildlife in a specific area is shaped by a blend of historical and continuous factors, this could involve factors such as commercial poaching, subsistence hunting, habitat degradation, fragmentation, ecological changes, and natural disruptions are factors at play. Local inhabitants with a longstanding presence in the region hold valuable knowledge regarding the spatial extent, level of impact, duration, and range of variations related to these processes^37^

The anticipated consequences of climate change are likely to affect a wide variety of organisms in ecosystems, with mammals, as indicated by studies by^38–40^ being one of the species expected to experience significant impacts. Conservation strategies, often reliant on distribution maps, are crafted for specific species. However, the creation of these maps, especially on a large spatial scale, presents substantial challenges, as highlighted by^41^. This underscores the pressing need for conservation measures aimed at safeguarding and preserving these species for the future. Additionally, it is noteworthy that nearly half of the species' original range has been lost due to various factors, emphasizing the urgency of addressing these threats and supporting robust conservation initiatives. Defining objectives for protected area planning in light of these challenges is a complex and multifaceted task.

Biodiversity spans a spectrum of ecological systems that cannot be adequately captured through a single parameter. Conservation planning inherently incorporates spatial aspects, making only mappable biodiversity characteristics pertinent to this procedure. The methods to map and quantify ecological and evolutionary processes are still in their nascent phases of advancement.^42^. In 1872, United States President Ulysses Grant took a momentous step by designating 2.2 million acres of wilderness as the inaugural formally acknowledged protected region, specifically Yellowstone National Park^43^. Initially established for recreational purposes, this marked the gradual inception of the concept of protected areas^44^. Over the following century, the significance of protected areas became widely acknowledged as indispensable for biodiversity conservation.

The reduction in both the size and number of protected areas underscores the urgent requirement to prioritize conservation initiatives and safeguard the habitats of the Asiatic black bear. Ensuring the preservation and expansion of these protected areas is essential to safeguard the species and contribute to overall biodiversity conservation. Many protected areas in Southeast Asia exhibit low mammal densities due to the impact of both commercial and subsistence hunting. Local communities contribute to this issue, but they can also play a crucial role in finding solutions through enhanced partnerships that involve their knowledge in identifying the problems. By engaging local people in the conservation process, a greater understanding of their positive and negative impacts on protected areas is fostered, leading to site-specific conservation assessments that inform effective management planning^45^

The importance of protected areas in conserving biodiversity is widely recognized. However, their creation and administration come with associated costs, encompassing expenses related to protection, management activities, and the foregone opportunities for local communities reliant on natural resource extraction. Thus, a meticulous and systematic approach is imperative when designing protected areas, aiming to minimize costs and maximize advantages. This entails establishing objectives for conserving a diverse range of attributes within protected areas, along with defining criteria to assess existing protected-area networks, steering their enhancement and growth^46^.

Wildlife conservation in South Asia is a difficult endeavor since it requires a balance between protecting natural resources and satisfying the expanding demands of the population and development. Wildlife scientists, managers, and policy makers must develop solutions for protecting threatened species and their ecosystems while also resolving conflicts between wildlife and people^15^

Wildlife scientists, conservationists, and policymakers face the critical task of devising strategies to safeguard endangered species and their natural habitats, all while mitigating conflicts that may arise between wildlife conservation and human interests^15^

## Conclusion

The current study reveals that the Asiatic black bear had much larger historical distribution range compared to its current one. However, the species range got substantially reduced since historical times (49.5% contraction). Similarly, approximately 35% of the protected areas have also lost the Asiatic black bear that they contained in historical times, indicating that even protected areas have not been successful in conserving this bear species. These findings urge and emphasize the managers the importance of implementing effective conservation efforts within the range of the Asiatic black bear.

### Supplementary Information

Supplementary Information.

## Data Availability

The datasets generated during and/or analyzed during the current study are available from the corresponding author on reasonable request.

## Abbreviations

CRS: Coordinate reference system
EN: Endangered
GIS: Geographic information systems
IUCN: International union for conservation of nature
KML: Keyhole markup language
PA’s: Protected areas
QGIS: Quantum geographic information systems
VU: Vulnerable
WGS: World geodetic system
WDPA: World database on protected area
